# New Onset of Acute and Chronic Hepatic Diseases Post-COVID-19 Infection: A Systematic Review

**DOI:** 10.3390/biomedicines12092065

**Published:** 2024-09-10

**Authors:** Ahamed Lebbe, Ali Aboulwafa, Nuran Bayraktar, Beshr Mushannen, Sama Ayoub, Shaunak Sarker, Marwan Nour Abdalla, Ibrahim Mohammed, Malik Mushannen, Lina Yagan, Dalia Zakaria

**Affiliations:** 1 Medical Department, Weill Cornell Medicine-Qatar, Doha 24144, Qatar; aaa4010@qatar-med.cornell.edu (A.L.); aaa4001@qatar-med.cornell.edu (A.A.); nub4001@qatar-med.cornell.edu (N.B.); sna4001@qatar-med.cornell.edu (S.A.); shs4020@qatar-med.cornell.edu (S.S.); msn4002@qatar-med.cornell.edu (M.N.A.); 2 Department of Medicine, Albany Medical College, New York, NY 12208, USA; mohammi3@amc.edu; 3 Department of Medicine, New York-Presbyterian Brooklyn Methodist Hospital, New York, NY 12208, USA; 4 Department of Medicine, University of Pennsylvania, Philadelphia, PA 19104, USA; 5 Premedical Department, Weill Cornell Medicine-Qatar, Doha 24144, Qatar

**Keywords:** COVID-19, SARS-CoV-2, post-COVID-19 sequelae, long-COVID, liver injury, hepatic injury, parenchymal liver disease

## Abstract

The SARS-CoV-2 virus caused a pandemic in the 2020s, which affected almost every aspect of life. As the world is recovering from the effect of the coronavirus, the concept of post-COVID-19 syndrome has emerged. Multiple organ systems have been implicated, including the liver. We aim to identify and analyze the reported cases of severe and long-term parenchymal liver injury post-COVID-19 infection. Several databases were used to conduct a comprehensive literature search to target studies reporting cases of severe and long-term parenchymal liver injury post-COVID-19 infection. Screening, data extraction, and cross checking were performed by two independent reviewers. Only 22 studies met our inclusion criteria. Our results revealed that liver steatosis, non-alcoholic fatty liver disease (NAFLD), and cirrhosis were the most reported liver associated complications post-COVID-19 infection. Moreover, complications like acute liver failure, hepatitis, and liver hemorrhage were also reported. The mechanism of liver injury post-COVID-19 infection is not fully understood. The leading proposed mechanisms include the involvement of the angiotensin-converting enzyme-2 (ACE-2) receptor expressed in the liver and the overall inflammatory state caused by COVID-19 infection. Future studies should incorporate longer follow-up periods, spanning several years, for better insight into the progression and management of such diseases.

## 1. Introduction

A new coronavirus emerged in 2019, causing a debilitating pandemic in 2020. The coronavirus was called severe acute respiratory syndrome coronavirus 2 (SARS-CoV-2), and the disease caused was called coronavirus disease 2019 (COVID-19). The infection ranged from asymptomatic and mild to severe, with infection of lung parenchyma and multiorgan dysfunction, often affecting those with comorbidities and underlying immunodeficiency [1].

As the world recovered from the pandemic, the concept of “long COVID” or post-acute sequelae of SARS-CoV-2 infection (PASC) and “post-COVID syndrome” came about as patients who recovered from the illness experienced a wide variety of symptoms. Some of the complications were short term. Symptoms occurring between 4 and 12 weeks after infection are referred to as post-COVID-19 condition, as per the Centers for Disease Control and Prevention (CDC), whereas long COVID typically refers to issues persisting after the 12-week period [2].

The SARS-CoV-2 virus affects many organs throughout the body, and the liver and biliary system have been implicated in long COVID manifestations [3]. SARS-CoV-2 is among many viruses that are associated with liver disease. Hepatitis associated with viral hemorrhagic fevers brought on by the likes of Dengue fever, as well as fulminant liver necrosis induced by the likes of adenoviruses, are just some examples of this [4]. Herpes viruses, in their unique manner, can remain dormant and reactivate at later stages of life, causing catastrophic disease ranging from fulminant hepatitis to lymphocyte proliferation and transformation into lymphoma [4]. Similar to SARS-CoV-2, respiratory viruses such as influenza viruses often display milder symptoms that more closely represent that of SARS-CoV-2. Many patients have abnormal liver chemistries post-COVID-19 infection; however, the clinical implications and long-term manifestations of liver injury inflicted by COVID-19 are not well defined in the literature. The liver injury observed is usually classified by deriving an R-factor suggestive of a hepatocellular or cholestatic pattern of injury. Parenchymal liver injury comprises a variety of lesions that include inflammatory infiltration, hepatocellular degenerative changes, and liver cell death via necrosis or apoptosis. Inflammatory infiltration is a common characteristic in cases of hepatitis and can sometimes go unnoticed through lab studies; hence, it is confirmed via imaging. Steatosis, bile stasis, ballooning, and Mallory–Denk bodies are just some features of hepatocellular degenerative changes observed in fatty liver diseases, such as NAFLD. A clear distinction between steatosis and steatohepatitis is important to observe in these cases, given the latter’s greater potential to progress to fibrosis and cirrhosis. These two characteristics can often be seen in conjunction, along with sclerosis as a long-term outcome. These characteristics often have a temporal relationship in parenchymal liver injury. Inflammation transitions to hepatocellular changes in response to the acute injury that may then progress gradually into cell death and its associated changes (fibrosis, sclerosis, etc.). Hepatocellular injury is specific to liver parenchyma, while cholestatic injury is reflective of injury that is biliary in origin. The degree of liver injury correlates to the severity of COVID-19 infection. Conversely, patients with pre-existing liver disease were noted to have an increased risk for severe COVID-19 infection and intensive care unit (ICU) admission [5]. Interestingly, non-alcoholic fatty liver disease (NAFLD) has been associated with a longer viral shedding time [5].

The objective of this systematic review is to look at the patterns and manifestations of parenchymal liver injury reported in individuals either after recovering from COVID-19 infection or those that occurred during the active infection and lasted and/or required treatment for 12 weeks or longer.

## 2. Materials and Methods

The preferred reporting items for systematic reviews and meta-analysis (PRISMA) statement was used to develop the protocol of this systematic review [6].

### 2.1. Information Sources and Search Strategy

This study is part of a large project that investigates the long-term and severe complications of COVID-19. A comprehensive search was conducted by an information professional who prioritized sensitivity to retrieve all relevant studies. The following databases were searched in October 2023: PubMed, Medline (Ovid, 1946–Current), Embase (Ovid, 1974–2021), Scopus, Web of Science, Science Direct, and Cochrane Library. The search was designed around keywords and controlled vocabulary that focused on “Long COVID” and variants (see Supplementary Materials S1 for full search details). No language or date restrictions were used. All database search results were imported into EndNote (version 19) and exported to Covidence, where duplicates were removed prior to initial screening (Supplementary Materials S1).

### 2.2. Eligibility Criteria

No restrictions were made based on gender, age, or country. Duplicates were removed and any articles that were not in English or did not have primary data, such as review articles, were excluded from the study. All conference abstracts were excluded, and only full articles were included. During the full-text screening, any studies that reported parenchymal liver injury were included if a clear diagnosis was reported. Any studies reporting only liver function test (LFT) derangement were excluded. We only included severe and/or long-term parenchymal liver complications. The inclusion criteria related to this point included any patients who developed a parenchymal liver injury after recovering from COVID-19. If the injury was diagnosed after at least a month post-COVID-19 diagnosis or if the study reported that anti-SARS-CoV-2 IgG but not IgM antibodies were detected, the study was included. The studies that reported parenchymal liver injury diagnosis during the active COVID-19 infection were included only if the patient did not recover from the liver injury within 12 weeks or required treatment for 12 weeks or more. The study was also included if the patient died within 12 weeks, which indicates severity. Any cases with parenchymal liver injury that were diagnosed during the active infection of COVID-19 and fully recovered within less than 12 weeks were excluded.

### 2.3. Study Selection and Data Collection

Title, abstract screening, and full-text screening were conducted by two independent reviewers for each study using Covidence. Disagreements were resolved by consensus. Data were extracted and cross checked by two independent reviewers.

### 2.4. Data Items

Demographic and clinical data, including age, sex, comorbidities, treatments, and outcomes, were collected. Continuous variables were expressed as mean ± standard deviation or range of results. Categorical variables were expressed as percentages.

### 2.5. Risk of Bias and Quality Assessment

The quality assessment was conducted using different methods depending on the type of study. The Newcastle–Ottawa Quality Assessment Scale (NOS) was used to assess the cohort studies [7]. The scale developed by Murad et al. was used to assess the case reports and case series [8]. Quality assessment was conducted and cross checked by two independent reviewers.

### 2.6. Data Analysis

The parenchymal liver disorders reported by the included studies were classified under the following categories: steatosis and NAFLD; liver fibrosis, sclerosis, and cirrhosis; hepatitis; acute liver failure; liver inflammation by imaging; hepatomegaly; and any other parenchymal liver disorders that do not fall under the above categories.

## 3. Results

**Figure 1:**
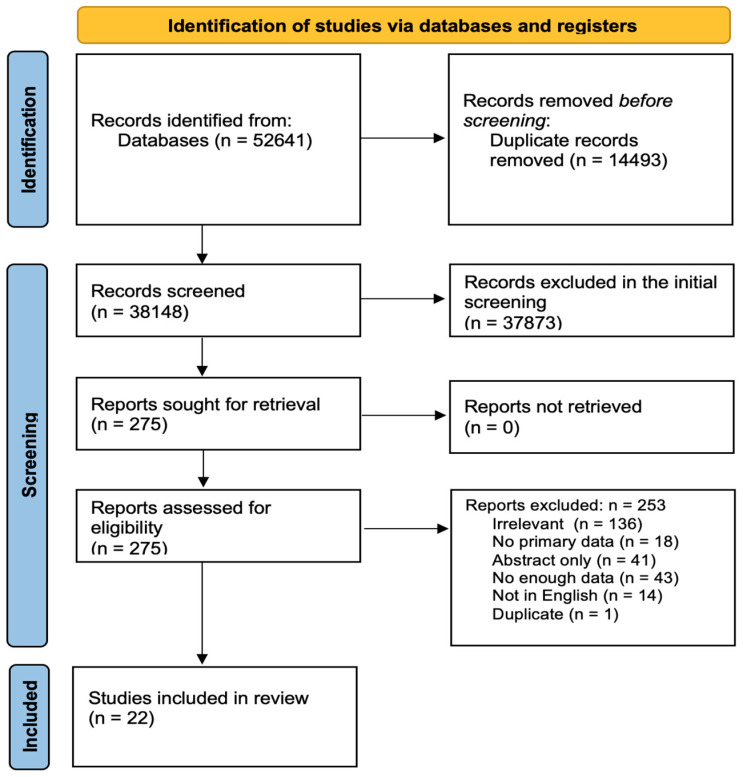
Protocol of database search, screening, and study selection.

Figure 1 shows the flow diagram of our protocol. After removing the duplicates, the titles and abstracts of 38148 studies were screened, of which 273 were selected for full-text screening. Only 22 studies met our inclusion criteria. Of the two hundred fifty-one excluded studies, one hundred thirty-four were irrelevant, eighteen had no primary data, forty-one were conference abstracts, forty-three did not have enough data, fourteen were not in English, and one was duplicate. Supplementary Table S1 summarizes the demographic and clinical data of the included subjects as well as the quality assessment score for each study [9,10,11,12,13,14,15,16,17,18,19,20,21,22,23,24,25,26,27,28,29,30].

### 3.1. Types of Studies and Demographic Data

Of the twenty-two included studies, nine were case reports, ten were cohort studies, one was a case series and one was a cross-sectional study. Among the twenty-two included studies, two were from the USA, three from India, four from the UK, two from Indonesia, two from Latvia, two from Romania, and one from Brazil, Israel, Taiwan, UAE, China, Hungary, and Italy each.

The total number of patients reported by 22 studies (case series and case reports) was 161,594. Of these patients, 500 sustained post-COVID-19 parenchymal liver disease. Approximately 48% of patients were males. Furthermore, the age of the included patients ranged from 3 months to 88 years (Supplementary Table S1).

### 3.2. Clinical Data

**Figure 2:**
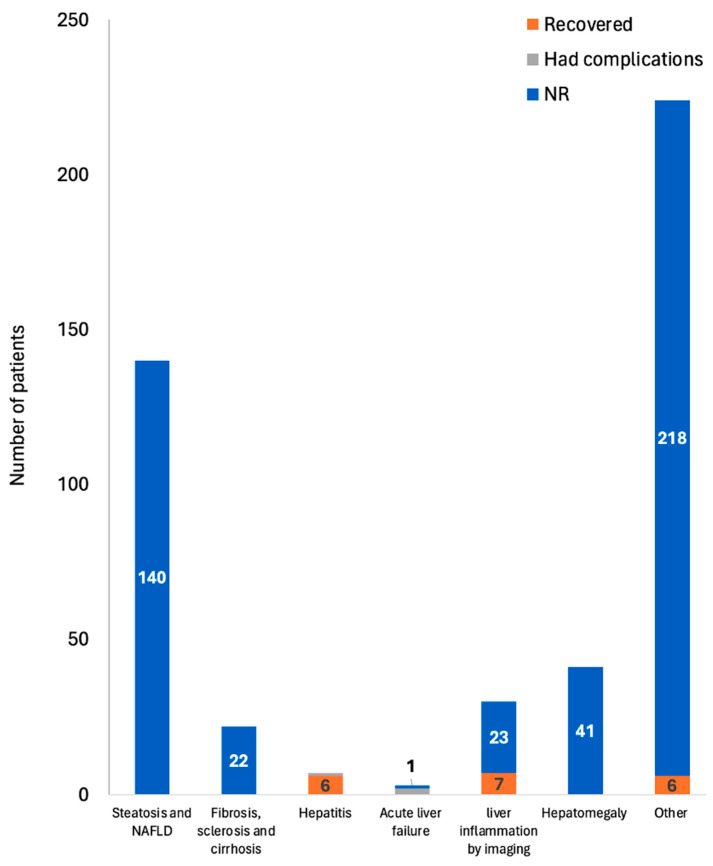
Types of the reported parenchymal liver injury post-COVID-19 infection and the reported outcomes. Only 1 death was reported by the 22 included studies. NAFLD: non-alcoholic fatty liver disease, NR: not reported.

The reported disorders related to the liver parenchyma post-COVID-19 were categorized into: steatosis and NAFLD, liver fibrosis, sclerosis, cirrhosis, acute liver failure, liver inflammation by imaging, hepatomegaly, hepatitis, and other post-COVID-19 parenchymal liver diseases that do not fall under the previous groups. An overlap exists, as certain studies reported various mechanisms of parenchymal liver injury. Therefore, some studies were included under multiple categories and some patients were counted under different categories (Supplementary Table S1). Figure 2 summarizes the types of reported parenchymal injury post-COVID-19 infection and the reported outcomes.

#### 3.2.1. Steatosis and NAFLD

Steatosis and NAFLD were reported by 7 studies on 140 patients whose age ranged from 52 to 74 years. This was excluding Ma et al., who reported that the hazard ratio (HR) of developing NAFLD was 1.33 (1.15 to 1.55 with *p* < 0.001) in patients who recovered from COVID-19 as compared to healthy controls.

**Figure 3:**
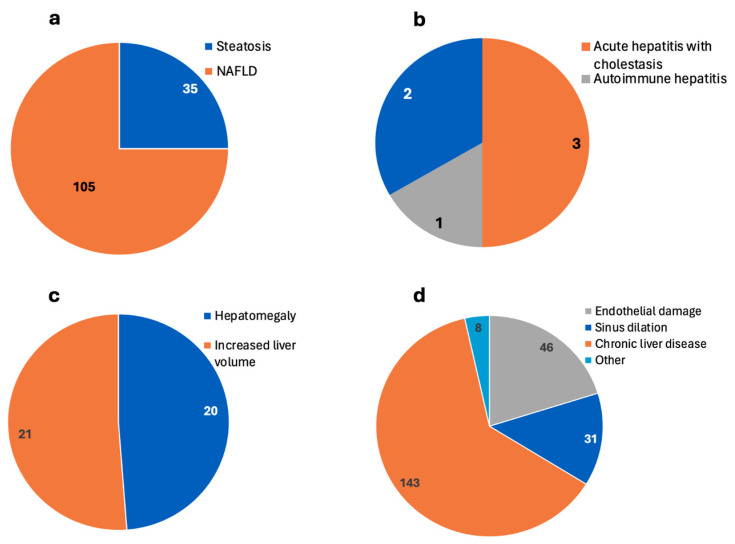
Types of post-COVID-19 parenchymal liver injury as reported by 22 included studies. The numbers in the figures show the reported number of patients. (**a**) Post-COVID-19 steatosis and NAFLD as reported by 7 included studies. (**b**) Post-COVID-19 acute hepatitis with cholestasis and autoimmune hepatitis as reported by 4 included studies. (**c**) Post-COVID-19 hepatomegaly and increased liver volume as reported by 2 included studies. (**d**) Post-COVID-19 endothelial damage, sinus dilation, chronic liver disease, or other disorders as reported by 11 included studies. The other disorders were reported only in 8 patients, such as autoimmune hemolytic anemia, acute liver injury with calcified liver nodule, portal venous thrombosis, liver abscesses, hemophagocytic lymphohistiocytosis, deranged LFTs with ascites, and congestive hepatopathy. Liver stiffness was also reported but the number of patients was not reported (NR). Liver fibrosis was reported in 7 patients while sclerosis and cirrhosis post-COVID-19 were reported without reporting the number of patients (4 studies). Acute liver failure was reported by 3 case reports and liver inflammation (by imaging) was reported in 29 patients by 2 studies. The last 3 categories were not presented as pie charts due to the lack of number of patients or because there were no different types to show on the pie chart. NAFLD: non-alcoholic fatty liver disease, NR: not reported.

Of the included 140 patients, 35 were reported to have steatosis, and 105 had NAFLD. The prevalence of steatosis among COVID-19 patients was 26% according to Roman et al. at the 6 month follow-up. Out of the 140 patients, 24 had severe COVID-19, but no deaths were reported (Table 1). Figure 3a summarizes the reported types of post-COVID-19 steatosis/NAFLD.

#### 3.2.2. Liver Fibrosis, Sclerosis, and Cirrhosis

Post-COVID-19 liver fibrosis, sclerosis, and cirrhosis were reported by four studies on 22 patients (Table 2).

#### 3.2.3. Hepatitis

Post-COVID-19 hepatitis was reported by four studies on six patients whose age ranged from 8 months to 25 years. Of the six patients, none of them were reported to have severe COVID-19, all six of them recovered from liver injury, and no deaths were reported (Table 3). Figure 3b summarizes the reported types of post-COVID-19 hepatitis.

#### 3.2.4. Acute Liver Failure

Acute liver failure was reported by three studies on three patients whose age ranged from 3 months to 65 years. Of the three patients, none of them were reported to have severe COVID-19, and two of them had remarkable complications of NAFLD and Hemophagocytic lymphohistiocytosis following the liver insult, but no deaths were reported (Table 4).

#### 3.2.5. Liver Inflammation by Imaging

Liver inflammation was reported by two studies on 29 patients whose age ranged from 56 to 88 years. Of the twenty-nine patients, seven of them were reported to have severe COVID-19 but no deaths were reported (Table 5).

#### 3.2.6. Hepatomegaly

Hepatomegaly was reported by two studies on 41 patients. Of the 41, 20 were reported to have hepatomegaly and 21 had increased liver volume by imaging. No deaths were reported (Table 6). Figure 3c summarizes the reported types of post-COVID-19 hepatomegaly.

#### 3.2.7. Other Parenchymal Liver Diseases

There were 11 further studies describing various other liver diseases experienced after COVID-19 in 225 patients (Table 7). Ayoubkhani et al. described 140 patients having unspecified chronic liver disease and reported that chronic liver disease was diagnosed in 0.3% of patients after discharge due to COVID-19 infection [9]. Daid et al. reported a case of intraparenchymal liver hemorrhage in a 43-year-old, and Liemarto et al. reported a case of liver hemorrhage with abscess and necrosis in a 49-year-old patient, who passed away on the 20th day of admission (Table 6 and Figure 3d) [18,24].

## 4. Discussion

Numerous studies have explored the hepatic manifestations associated with COVID-19 infection. This systematic review compiles data reporting parenchymal liver injury following COVID-19 infection to elucidate the underlying mechanisms, prevalence, and clinical implications of hepatic involvement as one of the severe and long-term complications of COVID-19.

This systematic review involves 22 studies from 13 different countries reporting 500 patients who had post-COVID-19 parenchymal liver injury. The most common manifestation was steatosis and NAFLD in addition to acute liver failure, hepatitis, fibrosis, and sclerosis.

### 4.1. Post-COVID-19 Steatosis and NAFLD

Non-alcoholic fatty liver disease (NAFLD), now referred to as metabolic dysfunction-associated steatotic liver disease (MASLD), encompasses a spectrum of liver conditions characterized by excessive fat accumulation in the liver without significant alcohol consumption or other secondary causes. NAFL is defined by hepatic steatosis without evidence of hepatocellular injury, such as ballooning degeneration of hepatocytes, while Nonalcoholic steatohepatitis (NASH) involves hepatic steatosis accompanied by inflammation and hepatocellular injury, which can progress to fibrosis, cirrhosis, and liver-related morbidity and mortality (Han S. et al.) [31]. The term MASLD was suggested to more accurately reflect the pathogenesis of the disease, with the diagnosis being made in the presence of hepatic steatosis and one of the following criteria: overweight/obesity, type 2 diabetes mellitus, and evidence of metabolic dysregulation [30]. The development of NAFLD is also influenced by genetic factors, with many studies having identified genetic drivers of NAFLD, beyond the established factors of metabolic syndrome and insulin resistance. Notably, the phospholipase domain-containing protein 3 (PNPLA3) and transmembrane 6 superfamily member 2 (TM6SF2) nucleotide polymorphisms were found to affect the development and progression of the disease [30]. Genes involved in carbohydrate and lipid metabolism, insulin signaling pathways, inflammatory pathways, oxidative stress, and fibrogenesis have also been linked with NAFLD/NASH development and progression [30]. Mitochondrial dysfunction is the key mediator triggering oxidative stress and is brought on by an imbalance of oxidants and antioxidants. Most notably, the uncoupling between β-oxidation, the tricarboxylic acid (TCA) cycle, and the electron transport chain (ETC) frequently results in inefficient lipid metabolism and reactive oxygen species (ROS) overproduction in the liver [32]. The capacity of antioxidant defense is further suppressed by ROS overproduction, leading to further oxidative damage [32].

Steatosis and NAFLD were among the most reported post-COVID-19 liver manifestations as reported by seven studies on 140 patients, of which 24 had severe COVID-19. Pesti et al. found that 15 of the 147 autopsies had steatosis [23]. However, a correlation between the severity of infection and the development of steatosis was not established by Pestii et al. [23]. However, Dennies et al. reported a more frequent incidence of steatosis in symptomatic groups [11]. Roman et al. reported that 26% of the patients had steatosis at the 6 month follow-up [25]. Furthermore, they reported that patients with severe COVID-19 were more likely to have persistent alanine transaminase (ALT) elevation at 6 months compared to those with non-severe COVID-19. Bende et al. investigated the outcomes of COVID-19 patients with and without pulmonary injury, delineating that pulmonary injury correlates with a severe disease course [27]. They found that patients with pulmonary injury were more likely to have steatosis. Ma et al. reported that the hazard ratio (HR) of developing post-COVID-19 NAFLD was 1.33 (CI 95%, 1.15 to 1.55 with *p* < 0.001) as compared to healthy controls [19]. Radzina et al. used attenuation imaging (ATI) to detect steatosis and found that post-COVID-19 individuals had significantly increased steatosis compared to healthy controls [22]. Milic et al. reported that 55% of patients had NAFLD on follow-up, while only 37% were calculated to have NAFLD during the initial admission [26]. This finding correlated to the weight changes during and after COVID-19 (–6 kg during, +5 kg after), leading the authors to suggest that the loss of muscle during the acute phase and a buildup of fat in the liver during the chronic/post-acute phase is what leads to NAFLD in individuals who recovered from COVID-19 infection.

A prominent theory regarding the mechanism of liver injury is that the angiotensin-converting enzyme-2 (ACE-2) receptor serves as a gateway for the virus. The spike protein on the virus surface binds to the ACE-2 receptor, facilitating this process. The ACE-2 receptor is reported to be expressed in hepatocytes and more frequently in cholangiocytes. Although there is not much evidence that the biliary tree is affected more than hepatocytes, interestingly, it is highly expressed in the endothelial layer of small blood vessels but is absent in the sinusoidal epithelium [33]. SARS-CoV-2 competes with Ang II for binding to ACE2, and when the virus binds, it inhibits ACE2 activity and decreases its expression on the cell membrane [34]. This can potentially disrupt the balance of the renin–angiotensin–aldosterone system (RAAS), leading to angiotensin-2 mediated vasoconstriction and a decrease in angiotensin (1-7)-mediated vasodilation that may contribute to inflammation [35]. Additionally, the kallikrein–kinin system (KKS), often considered an antagonist to the RAAS, mitigates inflammatory effects by reducing the generation of reactive oxygen species and lowering blood pressure [36]. The internalization of ACE-2 due to viral binding can further disrupt this balance by increasing bradykinin levels, which can activate the bradykinin (B) 2 receptor and enhance inflammation, particularly through the cytokine storm [37].

### 4.2. Post-COVID-19 Liver Fibrosis, Sclerosis, and Cirrhosis

Liver fibrosis occurs due to the excessive accumulation of extracellular matrix proteins, including collagen, in the liver, as a result of chronic liver injury or persistent inflammation [38]. Cirrhosis is the most advanced stage of liver fibrosis, where extensive scarring has disrupted the normal architecture of the liver, leading to the formation of nodules and impaired liver function. It is characterized by the presence of regenerative nodules surrounded by fibrous bands [39]. Our results revealed that three studies reported fibrosis and two studies reported cirrhosis as post-COVID-19 complications. Kolesova et al. reported that 65% of patients with acute COVID-19 exhibited an abnormal Fibrosis-4 Index (FIB-4), possibly serving as a derivative for systemic inflammation, hepatocellular damage, and infection severity [17]. FIB-4 scores may have been falsely elevated in the acute setting in the setting of inflammation, and a decrease in the index was observed post-recovery. In the post-COVID-19 group, 5% had increased levels of FIB-4. A history of liver disease did not influence FIB-4 in the post-COVID-19 group, suggesting that COVID-19 infection is the cause of liver fibrosis. Therefore, it is essential to monitor liver chemistries post-COVID-19 to facilitate early diagnosis and prevent liver damage [17]. Similarly, Pesti et al. reported fibrosis post-COVID-19 in seven patients out of one hundred fifty in an autopsy study [23]. Moreover, significantly increased fibrosis, viscosity, and steatosis were reported by Radzina et al. in post-COVID-19 patients compared to controls [22]. Finally, they reported a significantly positive relationship between ALT, gamma-glutamyl transferase (GGT), and body mass index (BMI) with liver fibrosis seen on shear wave dispersion (SWD).

Conversely, Pesti et al. suggested that fibrosis is likely due to pre-existing comorbidities or treatments rather than COVID-19 infections, as they found no association with the severity of COVID-19 and fibrosis or cirrhosis [23]. They observed signs of apoptosis in histopathology slides of the liver without detectable COVID-19 protein/RNA. This suggests that liver damage may occur indirectly due to other factors such as hypoxia caused by COVID-19 rather than through direct cytopathic mechanisms.

### 4.3. Post-COVID-19 Hepatitis

Hepatitis is inflammation of the liver, which can be caused by various factors such as viruses, heavy alcohol use, certain medications, and autoimmune disorders. This inflammation can lead to swelling and damage to the liver, affecting its function [40]. A total of six patients were reported to have hepatitis across four studies. Most patients were asymptomatic. Daga et al. described a patient with abdominal pain and nausea, who was later diagnosed with acute cholecystitis in addition to hepatitis [30]. Shorbagi et al. also reported a patient with abdominal pain and diarrhea who was found to have ulcerative colitis, potentially confounding the clinical picture [28]. However, it was later found that the patient had ulcerative colitis-like syndrome, which may have caused the symptoms. The patient was diagnosed with autoimmune hepatitis, identified by an elevated antinuclear antibody (ANA) and anti-smooth muscle antibody titers. Three patients were reported with cholestasis along with hepatitis by Cooper et al. and Dragenescu reported a case of isolated hepatitis in a 21-year-old [13,14]. All patients had mild COVID-19, which was supportively managed.

### 4.4. Post-COVID-19 Acute Liver Failure

Acute liver failure is characterized by the rapid deterioration of liver function, leading to the development of hepatic encephalopathy and impaired synthetic function. This often results in coagulopathy and altered mental status in individuals without preexisting liver disease [41]. Acute liver failure was reported by three studies on three patients, excluding Ma et al., who did not report data on liver failure [19]. Cooper et al. reported that two patients, one 3 months old and one 5 months old, required liver transplant [14]. The first case had a mild infection, but both cases presented with encephalopathy and deteriorated over the hospital course, requiring liver transplantation at the end. The case reported by Anguiano-Albarran et al. also presented with encephalopathy and renal failure [20]. The patient was treated with supportive measures for liver failure with fluid resuscitation and intravenous albumin. The patient recovered and was ultimately followed-up as a case of NAFLD.

It was proposed that the cytokine storm seen in critically ill COVID-19 patients is marked by an overactive immune response leading to biliary sclerotic alterations and liver dysfunction, which in turn contributes to multiorgan failure [20].

### 4.5. Post-COVID-19 Liver Inflammation by Imaging

Liver inflammation is a condition characterized by the swelling and damage of liver tissue. This inflammation is the body’s natural response to injury or infection [42]. Two studies reported 29 patients with liver inflammation on imaging via positron emission tomography (PET) scan: Bai et al., with magnetic resonance imaging (MRI), and Dennis et al. [10,11]. The imaging, conducted 16 days on average after discharge, showed that post-COVID-19 patients had significantly higher standardized uptake values (SUV)max and (SUV)avg compared to controls (*p* < 0.05) (Bai et al.). All patients had severe COVID-19, in addition to evidence of inflammation in various other organs. No follow-up data were reported by this study. On the other hand, Dennis et al. followed the patients over 4 months and showed that liver inflammation was detected to a greater extent in post-COVID-19 patients than controls. Furthermore, they reported that liver inflammation and fibrosis (referred to as fibro inflammation together) were associated with cognitive dysfunction.

### 4.6. Post-COVID-19 Hepatomegaly

Hepatomegaly is a physical finding that may suggest intrinsic liver dysfunction and is a non-specific finding in many liver-related conditions [43]. Hepatomegaly was reported by two studies on 41 patients. Dennis et al. reported increased liver volume in 10.4% of the patients who were followed after COVID-19 infection, which was significantly higher than the control group (*p* < 0.0001) [11]. Furthermore, Dennis et al. reported hepatomegaly in 20 of the patients in the follow-up cohort, and it was associated with a poor quality of life [12]. However, no specifics were mentioned regarding other symptoms or impairments. The study also mentioned that liver impairment was more common in obese individuals and women.

### 4.7. Other Post-COVID-19 Parenchymal Liver Disorders

Congestive hepatopathy is a liver disorder that occurs due to prolonged passive venous congestion of the liver, commonly because of right-sided heart failure [44]. Congestive hepatopathy was reported in one patient by Ahmed et al.; the patient was described to have dilated cardiomyopathy 2 weeks after his COVID-19 infection diagnosis with deranged liver chemistries, which resolved after diuresis [15]. This was attributed to either COVID-19 or drug-induced liver injury (DILI) from herbal medicine, which the patient took prior to admission. Deranged LFTs are very common following viral infections and are non-specific. Lai et al. reported a case of deranged LFTs with a calcified liver nodule seen on imaging, while Sai et al. reported a case of deranged LFTs and ascites [16,29]. However, the clinical significance and outcome were not clearly defined.

Two cases reported liver hemorrhage post-COVID-19 infection. Daid et al. described a patient who developed right upper quadrant (RUQ) pain and abnormal liver chemistries around 2 weeks after her admission for COVID-19 infection, which was treated with N-acetylcysteine [18]. The mechanism of injury was believed to involve endothelial cell damage caused by widespread vascular thrombosis accompanied by microangiopathy, as demonstrated in earlier studies [45,46]. The study suggests that physicians should consider intraparenchymal hepatic hemorrhage when encountering a patient who recovered from COVID-19 and is presenting with RUQ pain and elevated transaminase levels.

Hepatomegaly was reported by Liemarto et al. in a 49-year-old patient who presented, a month after his COVID-19 infection recovery, with abdominal pain and hepatomegaly [24]. It was found that the patient had two abscesses with hemorrhage, which were drained via an exploratory laparotomy (ex-lap). The study suggests many etiologies for liver damage after COVID-19 infection, including direct damage from SARS-CoV-2 proliferation in the liver, systemic changes due to inflammation, hypoxia, and microvascular damage [47]. It was also suggested to be drug-induced, as the patient received tocilizumab, which has been reported to cause liver injury through the IL-6 signaling relating to liver recovery [48].

Soldera et al. described a case of Hemophagocytic Lymphohistiocytosis (HLH) after COVID-19 infection [21]. HLH is a rare but severe systemic inflammatory syndrome characterized by excessive immune activation and an uncontrolled proliferation of activated lymphocytes and macrophages. This condition leads to widespread inflammation and tissue damage, affecting multiple organs [49]. The patient presented with high-grade fever, upper abdominal pain, and transaminitis. Bone marrow biopsy confirmed HLH, and it was successfully treated with etoposide, dexamethasone, and cyclosporine. Soldera et al. emphasized that severe cases of COVID-19 can result in liver injury, potentially leading to the development of HLH [21]. It is recommended to assess patients with post-COVID-19 liver injury for HLH, particularly if they exhibit a high-grade fever and have a history of rheumatic diseases. Prompt diagnosis and treatment of HLH following a COVID-19 infection are essential to avoid adverse outcomes [21].

A case of cold autoimmune hemolytic anemia was reported by Daga et al. a month after the resolution of the patient’s COVID-19 infection [30]. Of note, the patient also had acute icteric hepatitis. The hypothesized mechanism involves post-infectious impact of COVID-19 causing a viral reactivation, as the patient later tested positive for Epstein–Barr virus (EBV) IgG and cytomegalovirus (CMV) IgG titers. The presence of these antibodies, along with the autoimmune hemolytic anemia and the absence of other hepatic insults, suggests a possible relationship between the post-COVID inflammatory phase and latent viral pathogens.

An association between pulmonary injury and liver stiffness was reported by Bende et al. [27]; it was revealed that patients who had pulmonary injury had significantly higher liver stiffness measured via liver elastography after 3 to 11 weeks following the subsidization of symptoms from COVID-19 infection. Therefore, it was concluded that it may be beneficial to routinely investigate patients within 12 weeks following COVID-19 infection to detect early signs of fibrosis and prevent progression.

Interestingly, Ayoubkhani et al. looked at rates of chronic liver disease after COVID-19 infection and found that chronic liver disease was diagnosed in 0.3% of patients after discharge, and this was 2.8 times more frequently diagnosed than controls [9]. However, the types of liver disease were not reported.

### 4.8. Study Limitations

The limitations of this study included the possible overlap between the categories of the parenchymal liver disorders and between the hepatobiliary and parenchymal injury. Patients with multiple types of injury were reported by some studies. These patients were clustered without describing the individual cases. Therefore, some patients were counted multiple times under different categories due to the inability to specify which patient had which combination of disorders. The small number of the included studies is another limitation, which is attributed to excluding conference abstracts. Only full article journal publications were selected to maintain the quality of the included studies and to avoid duplication when the work is published in both conferences and journals. Furthermore, another limitation was the small number of cohort studies as most of the included studies were either case series or case reports. This did not provide enough data to calculate the rate of incidence of parenchymal liver injury post-COVID-19 infection. However, the case reports provided enough details about the individual cases, which gave a better insight into the included cases with more details about prognosis and management.

## 5. Conclusions

This systematic review looked at the acute and chronic parenchymal liver complications of COVID-19. Common chronic complications that were found in patients’ weeks after their COVID-19 infection included liver steatosis, fibrosis, NAFLD, and chronic liver disease. Complications like hepatitis, acute liver failure, and hemorrhage were observed shortly after recovery. Most of the patients were described as recovered or recovering from liver injury. However, two patients with acute liver failure had to undergo liver transplants, and one patient with liver abscess and hemorrhage died. The mechanism of SARS-CoV-2’s direct viral attack affecting the liver centers around the expression of the ACE-2 receptor by the hepatocytes. The indirect mechanisms include the overall inflammatory state of COVID19 infection, drug side effects, and previous comorbidities. The acute complications are usually severe and should be monitored for patients who recently recovered from COVID-19. Chronic complications, like steatosis, fibrosis, and cirrhosis are typically not as symptomatic; thus, it is important for clinicians to monitor patients for these complications. The association between vaccination status and long COVID-19 liver injury is unclear, mostly due to underreporting of vaccination status in the studies. The connection between the severity of COVID-19 and the risk of long COVID-19-related liver injury is not well documented in most studies. Milic et al. found that 18% of patients who developed NAFLD required invasive ventilation, compared to 20% of those who did not develop NAFLD. Further research is necessary to determine whether the severity of COVID-19 influences the likelihood of long-term liver complications. Further studies should focus on how comorbidities and factors surrounding the COVID-19 infection influence the development of liver complications following infection. To gain a more comprehensive understanding of liver injury in post-COVID-19 patients, future studies should incorporate longer follow-up periods, spanning several years after recovering from COVID-19.

## Figures and Tables

**Table 1:** Post-COVID-19 steatosis and NAFLD.

Study	Study Type/County	N (Total)/Gender (%M)	n/N (%)/Type of LPD	* Age (Years)	Previous Liver Disease	COVID-19 Severity	Follow-Up Time/Outcome
[11]	Prospective longitudinal study/UK	Baseline 536/(27%M)	NR Liver steatosis	NR	NR	13% hospitalized	196 (182–209) days
		Follow-up (over 1 year) 331/(27%M)	NR	NR	NR		NA
[19] ^	Large-scale prospective population cohort study/UK	COVID-19 group 112,311/(45.2%M)	NR NAFLD	NR	NR	Non-hospitalized Hospitalized Severe	254 (IQR 184–366) days
		Contemporary control group 359,671/(44.6%M)	NR NAFLD	NR	NR	NR	254 (184–366) days
		Historical control group 370,979/(55%M)	NAFLD	56.94	NR	NR	254 (IQR 184–367) days
[22]	Observational cohort study/Latvia	Post-COVID-19 56/(50%M)	NR Steatosis	NR	NR	Moderate: 10/30 (33%) Severe: 7/30 (23%)	NR
		Control 34/(38%M)	NR Steatosis Fibrosis	NR	NR	NR	NR
[23] ^	Observational cohort study/Hungary	150/(54%M)	99/(66%) Steatosis (n = 147): F3: 15 F2: 29 F1: 49 F0: 54	NR	NR	83 ICU	NR
[25] ^	Prospective cohort study/Romania	78/(56%M)	58 (74%) Hepatic steatosis: 20/(26%)	NR	NR	NR 60 non-severe 18 severe	6 months
[26]	Retrospective cohort study/Italy	235/(68.9%M)	105/(77%) NAFLD	61 (52–72.5)	None	Patients with IV/NIV: 45	NR
		105/(63.8%M) No NAFLD at follow-up	NR Median reported PACS symptoms: 3	63 (52–74)	None	Patients with IV/NIV: 21 (*p* = 0.9)	As above
		130/(73.1%M) NAFLD at follow-up	NR ** NAFLD	60 (52–70)		patients with IV/NIV: 24	As above
[27]	Prospective cohort study/Romania	Pulmonary injury group 53/(43.4%M)	NR Steatosis	NR	None	NR	NR
		Non-pulmonary injury group 44/(31.8%M)	NR	NR	None	NR	NR

ICU: intensive care unit, IV: invasive ventilation, LPD: liver parenchymal disease, M: male, NIV: noninvasive ventilation, NAFLD: non-alcoholic fatty liver disease, NR: not reported. * Mean ± SE/Median (IQR), ** Criteria: Presence of liver steatosis (score > 36 on hepatic steatosis index) + body mass index (BMI) > 25 or type 2 diabetes militis (T2DM). Median reported PACS system: 2. ^ Includes patients with multiple types of parenchymal liver injury.

**Table 2:** Post-COVID-19 liver fibrosis, sclerosis, and cirrhosis.

Study	Study Type/County	N (Total)/Gender (%M)	n/N (%)/Type of LPD	* Age (Years)	Previous Liver Disease	COVID-19 Severity	Follow-Up Time/Outcome
[17]	Cross-sectional, single-center study/Latvia	Acute COVID-19 group 66/(50%M)	NR Liver fibrosis	NR	NR	NR	No follow-up
		Post-COVID-19 group 58/(53%M)	NR Liver fibrosis	NR	NR	NR	No follow-up
		Control group 17/(53%M)	NR	NR	NR	NR	No follow-up
[19] ^	Large-scale prospective population-based cohort study/UK	COVID-19 group 112,311/(45.2%M)	NR Hepatic sclerosis or cirrhosis	NR	NR	Non-hospitalized Hospitalized Severe	254 (IQR 184–366) days
		Contemporary control group 359,671/(44.6%M)	NR Hepatic sclerosis or cirrhosis	NR	NR	NR	254 (184–366) days
		Historical control group 370,979/(55%M)	Hepatic sclerosis or cirrhosis	56.94	NR	NR	254 (IQR 184–367) days
[22]	Observational cohort study/Latvia	Post- COVID-19 56/(50%M)	NR Fibrosis	NR	NR	Moderate: 10/30 (33%) Severe: 7/30 (23%)	NR
		Control 34/(38%M)	NR Fibrosis	NR	NR	NR	NR
[23]	Observational cohort study/Hungary	150/(54%M)	99 (66%) Cirrhosis F4 fibrosis: 7 F3 fibrosis: 3 F1/2 fibrosis: 68 No fibrosis/F0: 72	NR	NR	83 cases ICU	NR

ICU: intensive care unit, LPD: liver parenchymal disease, M: male, NR: not reported. * Mean ± SE/Median (IQR). ^ Includes patients with multiple types of parenchymal liver injury.

**Table 3:** Post-COVID-19 hepatitis.

Study	Study Type/County	N (Total)/Gender (%M)	n/N (%)/Type of LPD	* Age (Years)	Previous Liver Disease	COVID-19 Severity	Follow-Up Time/Outcome
[13]	Case report/Romania	1 M	1/(100%) Immune hepatitis	21	None	Mild COVID-19	ALT normalization 20 days after peak on follow-up
[14]	Retrospective case series/Israel	5/(100%M)					
			Patient 3: Acute hepatitis with cholestasis	8	None	Mild	4 months
			Patient 4: Acute hepatitis with cholestasis	8	NAFLD	Mild	4 months
			Patient 5: Acute hepatitis with cholestasis	13	None	Mild	45 days
[27]	Case report/UAE	1/(100%M)	1/(100%) Autoimmune hepatitis	33	None	Mild	7 months after hospitalization (asymptomatic)
[30]	Case report/India	1 F	1/(100%) Acute hepatitis	25	NR	Mild	5 months after presentation

ALT: alanine transaminase, F: female, LPD: liver parenchymal disease, M: male, NR: not reported. * Mean ± SE/Median (IQR).

**Table 4:** Post-COVID-19 acute liver failure.

Study	Study Type/County	N (Total)/Gender (%M)	n/N (%)/Type of LPD	* Age (Years)	Previous Liver Disease	COVID-19 Severity	Follow-Up Time/Outcome
[14]	Retrospective case series/Israel	5/(100%M)	5/(100%) Patient 1: Acute liver failure	Patient 1: 3-month-old	None	Mild	NR
			Patient 2: Acute liver failure	Patient 2: 5-month-old	None	NR	NR
[19] ^	Large-scale prospective population-based cohort study/UK	COVID-19 group 112,311/(45.2%M)	NR Liver failure	NR	NR	Non-hospitalized Hospitalized Severe	254 (IQR 184–366) days
		Contemporary control group 359,671/(44.6%M)	NR Liver failure	NR	NR	NR	254 (184–366) days
		Historical control group 370,979/(55%M)	Liver failure	56.94	NR	NR	254 (IQR 184–367) days
[20]	Case report/USA	1 F	1 (100%) Acute liver failure	68		NR	NR

F: female, LPD: liver parenchymal disease, M: male, NR: not reported. * Mean ± SE/Median (IQR). ^ Includes patients with multiple types of parenchymal liver injury.

**Table 5:** Post-COVID-19 liver inflammation by imaging.

Study	Study Type/County	N (Total)/Gender (%M)	n/N (%)/Type of LPD	* Age (Years)	Previous Liver Disease	COVID-19 Severity	Follow-Up Time/Outcome
[10] ^	Prospective study/China	COVID-19 cohort 7/(42%M)	7/(100%) ** Liver inflammation by imaging	66 (56–88)	HLD: 0/7 (0%)	Severe	16.1 days after discharge (on average)
[12] ^	Prospective observational cohort study/UK	Experimental cohort (post-COVID-19 syndrome patients) 201/(29.4%M)	56/(28%) Increased liver inflammation (≥784 ms cT1) 11.5%	NR	NR	NR 19% hospitalized	NR
		Control cohort (healthy individuals) 36/(88.6%M)	NR Increased Liver inflammation (≥784 ms cT1) 0%	NR	NR	NR	NR

HLD: Hyperlipidemia, M: male, NR: not reported. * Mean ± SE/Median (IQR). ** SUVavg, SUVmax in liver—significantly higher (*p* < 0.05) vs. controls. No significant difference b/w CTmax and CTavg vs. controls. LPD: liver parenchymal disease. ^ Includes patients with multiple types of parenchymal liver injury.

**Table 6:** Post-COVID-19 hepatomegaly.

Study	Study Type/County	N (Total)/Gender (%M)	n/N (%)/Type of LPD	* Age (Years)	Previous Liver Disease	COVID-19 Severity	Follow-Up Time/Outcome
[11] ^	Prospective longitudinal study/UK	Baseline 536/(27%M)	NR	NR	NR	13% hospitalized due to COVID-19	196 (182–209) days
		Follow-up (over 1 year) 331/(27%M)	20/(14%) Hepatomegaly	NR	NR		NR
[12] ^	Prospective observational cohort study/UK	Experimental cohort (post-COVID-19 syndrome patients) 201/(29.4%M)	56/(28%) Increased liver volume (≥1935 mL) 10.4%	NR	NR	NR 19% of patients hospitalized	NR
		Control cohort (healthy individuals) 36/(88.6%M)	NR Increased liver volume (≥1935 mL) 2.9%	NR	NR	NR	NR

LPD: liver parenchymal disease, M: male, NR: not reported. * Mean ± SE/Median (IQR). ^ Includes patients with multiple types of parenchymal liver injury.

**Table 7:** Other parenchymal liver post-COVID-19 complications.

Study	Study Type/County	N (Total)/Gender (%M)	n/N (%)/Type of HPD	* Age (Years)	Previous Liver Disease	COVID-19 Severity	Follow-Up Time/Outcome
[9]	Retrospective cohort study/UK	Experimental group (COVID-19 group) 47,780/(54.9%M)	143/(0.3%) Chronic liver disease NR	NR	NR	10% of patients needed ICU	Mean follow-up 140 ± 50 days Maximum 253 days
		Matched control group (individuals who did not test positive for COVID-19) 47,780/(54.9%M)	NR	NR	NR	NR	Mean follow-up 153 ± 33 days Maximum 253 days
[15]	Case study/India	1/(100%M)	Congestive hepatopathy	17	None	Mild COVID-19	3 months Discharged
[16]	Single-center prospective observational study/India	78/(34.6%M)	NR Deranged LFTs: 40/78 Ascites: 1/78	NR	NR	NR	No follow-up
[18]	Case study/USA	1 F	100% Intraparenchymal liver hemorrhage	43		Severe COVID-19 infection	Until discharge only
[21]	Case report/Brazil	1 M	1/(100%) hemophagocytic lymph histiocytosis	57	None	Mild COVID-19 infection	NR
[23] ^	Observational cohort study/Hungary	150/(54%M)	99/(66%) Endothelial damage (n = 119): F3: 46 F2: 52 F1: 21 F0: 0 Sinus dilatation (n = 119): F3: 31 F2: 52 F1: 36 F0: 0	NR	NR	83 cases requiring ICU	NR
[24]	Case report/Indonesia	1 M	1/(100%) Liver abscesses with hemorrhage and parenchymal necrosis	49	None	Severe COVID-19 pneumonia admitted to ICU	Died on day 20
[25] ^	Prospective cohort study/Romania	78/(56%M)	58/(74%) Portal venous system thrombosis: 2 (2.5%)	NR	NR	NR 60 non-severe 18 severe	6 months
[27] ^	Prospective cohort study/Romania	Pulmonary injury group 53/(43.4%M)	NR Liver stiffness	NR	None	NR	NR
		Non-pulmonary injury group 44/(31.8%M)	NR Liver stiffness	NR	None	NR	NR
[29]	Case report/Taiwan	1 F	1/(100%) Acute liver injury + calcified liver nodule	60	None	Mild	Over 3 months, LFTs trended down until returning to normal by the last follow-up visit
[30]	Case report/India	1 F	1/(100%) Autoimmune hemolytic anemia	25	NR	Mild	5 months after presentation

F: female, ICU: intensive care unit, LFTs: liver function tests, M: male, NR: not reported. * Mean ± SE/Median (IQR). ^ Includes patients with multiple types of parenchymal liver injury.

## Data Availability

All extracted data for this systematic review are submitted as Supplementary Materials.

## Supplementary Materials

The following supporting information can be downloaded at https://www.mdpi.com/article/10.3390/biomedicines12092065/s1, Table S1: Demographic and clinical data for patients reported with parenchymal liver injury reported post-COVID-19 infection.; Supplementary S1: Database search strategy.
