# Supplementary material for: New Onset of Acute and Chronic Hepatic Diseases Post-COVID-19 Infection: A Systematic Review
Source: Biomedicines. 2024 Sep 10;12(9):2065. doi: 10.3390/biomedicines12092065 (PMC11428502; doi:10.3390/biomedicines12092065)
Supplement: Supplementary file 1 [file biomedicines-12-02065-s001.zip › Supplementary Table S1.pdf]

Supplementary Table S1: Demographic and clinical data for patients reported with parenchymal liver injury reported post-COVID-19 infection.

Author	Study type<br>County	N (total)<br>Gender (%M)	n/N (%)<br>Type of PLD<br>Clinical features	Age Mean ±SE/Median (IQR) (years)	Comorbidities (before COVID-19)	COVID-19 Date/duration/Severity	Time of PLD diagnosis	Blood markers	Management	Other complications	Follow-up time Outcome	QA score Scale name
Ayoubkhani et al. 2021 [9]	Retrospective cohort study<br>UK	Experimental group (COVID-19 group) 47,780 (54.9% M)	143 (0.3%)<br>Chronic liver disease<br>NR	NR	NR	COVID-19 diagnosis 1 Jan to 31 Aug, 2020<br>4745 (10%) required ICU<br>43035 (90%) did not require ICU	After hospital discharge<br>Jan 1, 2020 - 31 Aug, 2020	NR	NR	NR	Mean follow-up-140±50 days<br>Maximum 253 days	8<br>NOS
		Matched control group (individuals who did not test positive for COVID-19) 47,780 (54.9% M)	NR	NR	NR	NA	After hospital discharge<br>Before 31 Aug, 2020	NR	NR	NR	Mean follow-up-153±33 days<br>Maximum 253 days	
Bai et al. 2021 [10]	Prospective study<br>China	COVID-19 cohort 7 (42% M)	7 (100%)<br>SUVavg	66 (56-88)	HTN: 2/7 (29%)<br>CAD: 2/7 (29%)	Jan 20- Feb 7, 2020	PET scan 25.3±6.3	ALT: 27.7±16.6 U/L	NR	NR	16.1 days after discharge	7<br>NOS

			SUVmax in liver significantly higher ($p < 0.05$) vs. controls<br>No significant difference b/w CTmax and CTavg vs. controls		COPD: 1/7 (14%)<br>DM: 0/7 (0%)<br>HLD: 0/7 (0%)	Severe COVID-19	days after admission<br><br>Tests performed once PCR tests were negative	AST: 28.2±6.0 U/L			(on average)	
Dennis et al. 2021 [11]	Prospective longitudinal study<br><br>UK	Baseline 536 (27%)	NR<br><br>Liver steatosis (more frequent in symptomatic groups)	NR	NR	497/536 COVID-19 exposure Jan-Sep, 2020<br><br>39/536 COVID-19 exposure after Sep, 2020<br><br>13% hospitalized due to COVID-19	182 (132-222) days from initial COVID-19 symptoms (during baseline assessment)	High ALT: 74/508<br><br>High albumin: 27/508<br><br>High ALP 13/508<br><br>High AST: 43/488<br><br>High bilirubin: 17/508<br><br>High cholesterol: 236/508<br><br>High GGT: 32/508<br><br>Low HDL: 40/508	NR	NR	196 (182-209) days	5<br><br>NOS

							High LDL: 167/500<br><br>High triglycerides: 71/508<br><br>High transferrin sat: 9/501<br>Low transferiin sat: 79/501			
	Follow-up (over 1 year)<br>331 (27%)	20 (14%)<br><br>Hepatomegaly: 20/138 with poor QoL 8/167 with better QoL<br><br>High liver fibro inflammation associated with cognitive dysfunction	NR	NR		196 (182-209) days after baseline assessment	High ALT: 46/326<br><br>High albumin: 11/326<br><br>High ALP: 11/326<br><br>High AST: 37/312<br><br>High bilirubin: 12/326<br><br>High cholesterol: 157/326<br><br>High GGT: 18/326<br><br>Low HDL: 31/326	NR	NR	NA

								High LDL: 116/325<br><br>High triglycerides: 44/326<br><br>High transferrin sat: 6/319<br><br>Low transferrin sat: 65/319				
Dennis et al. 2021 [12]	Prospective observational cohort study<br><br>UK	Experimental cohort (post-COVID-19 syndrome patients) 201 (29.4%M)	56 (28%)<br><br>Increased liver inflammation (≥ 784 ms cT1) 11.5%<br>Elevated liver fat(≥4.8%) 11.5%<br><br>Increased liver volume (≥ 1935 mL) 10.4%	NR	NR	NR<br><br>Assessments performed median 141 (110-162) days after initial COVID-19 infection 37/200 patients hospitalized for COVID-19 symptoms	Median 141 (110-162) days after initial COVID-19 infection	High bilirubin: (>20 U/L) 1.7%<br><br>Abnormal low ALP (< 40 IU/L in men; < 35 IU/L in women) 4.5%<br><br>Abnormal high ALP(> 129 IU/L in men; > 104 IU/L in women)<br><br>Abnormal high AST (> 129 IU/L in	NR	NR	NA	4<br><br>NOS

men; > 104 IU/L in women) 6.9%
Abnormal low ALT(< 10 IU/L in men; < 10 IU/L in women) 1.1%
Abnormal high ALT(> 50 IU/L in men; > 35 IU/L in women) 14%
Abnormal low GGT(< 10 IU/L in men; < 6 IU/L in women) 2.2%
Abnormal high GGT(> 71 IU/L in men; > 42 IU/L in women) 5.1%

								Low albu-min(<34g/L) 0%<br><br>High Albu-min (>50g/L) 6.2%				
		Control co-hort (healthy individu-als)<br>36 (88.6% M)	NR<br><br>Increased Liver in-flamma-tion (≥ 784 ms cT1) 0%<br>Elevated liver fat (≥ 4.8%) 5.4%<br><br>Increased liver vol-ume (≥ 1935 mL) 2.9%	NR	NR	NA	NA	NR	NA	NA	NA	
Drăgăn-escu et al. 2021 [13]	Case report<br><br>Romania	1 M	1 (100%)<br><br>Immune hepatitis<br><br>LFT de-rangement otherwise asympto-matic	21	None	June, 2020<br><br>Mild COVID-19<br><br>Negative PCR 16-17 days after admission<br><br>Discharged after 27 days	20 days after full remis-sion of COVID-19	Max. ALT: 736 U/L<br><br>Max. AST: 290 U/L<br><br>CBC<br>TBili<br>DBili<br>GGT<br>Fibrinogen<br>Coagulation markers all	Hospitalized with close monitoring	NA	ALT nor-malization 20 days af-ter peak on follow-up	6<br><br>Murad et al.

								within reference range				
Cooper et al. 2022 [14]	Retrospective case series<br><br>Israel	5 (100% M)	5 (100%)<br><br>Patient 1: Acute liver failure<br><br>Progressive jaundice	Patient 1: 3-month-old	Patient 1: None	Patient 1: February, 2021<br><br>Mild	Patient 1: 21 days after COVID-19 diagnosis	Patient 1:<br>AST: 2078 IU/L<br><br>ALT: 1440 IU/L<br><br>ALP: 2042 IU/L<br><br>GGT: 63 IU/L<br><br>TBili: 18.5 mg/dl<br><br>DBili: 14.5 mg/dl<br><br>Albumin: 4 g/dL<br><br>Ammonia: 184 μg/ dL<br><br>INR: 5.5	Patient 1: Vitamin K (coagulopathy did not resolve)<br><br>Liver transplant (on day 32) due to deteriorating liver synthetic function	Patient 1: Encephalopathy	Patient 1: NR	5<br><br>Murad et al.
			Patient 2: Acute liver failure<br><br>Jaundice; Dark urine; Acholic soft stools;	Patient 2: 5-month-old	Patient 2: Hypospadias<br><br>GERD	Patient 2: May, 2021<br><br>severity NR	Patient 2: At the time of presentation to the ED	Patient 2:<br>AST: 2265 IU/L<br><br>ALT: 2219 IU/L<br><br>ALP: 1034 IU/L	Patient 2: Liver transplant (on day 7) as INR continued to rise with clinical deterioration	Patient 2: Secondary hemophagocytic lymph histiocytosis (HLH) with cytopenia	Patient 2: NR	

		Hepatomegaly; Fever					GGT: 124 IU/L<br><br>TBili: 7.7 mg/dl<br><br>DBili: 4.5 mg/dl<br><br>Albumin: 4.3 g/dL<br><br>Ammonia: 110 µg/ dL<br><br>INR: 1.85 (after vitamin K)		High ferritin level<br><br>Hypofibrinogenemia<br><br>Elevated IL-2 levels<br><br>Hemophagocytes on liver histology<br><br>Encephalopathy<br><br>Post-operative infection with CMV and Adenovirus	
		Patient 3: Acute hepatitis with cholestasis<br><br>Abdominal pain; Vomiting; Jaundice; Hepatomegaly	Patient 3: 8	Patient 3: None	Patient 3: December 2020<br><br>Mild	Patient 3: 130 days after COVID-19 diagnosis	Patient 3: AST: 3598 IU/L<br><br>ALT: 3561 IU/L<br><br>ALP: 496 IU/L<br><br>GGT: 167 IU/L	Patient 3: IV and oral steroids	Patient 3: None	Patient 3: 4 months

						TBili: 8.1 mg/dl<br><br>DBili: 5.1 mg/dl<br><br>Albumin: 4.2 g/dL<br><br>Ammonia: 50 µg/ dL<br><br>INR: 1.5 (after vitamin K)			
	Patient 4: Acute hepatitis with cholestasis<br><br>Fever; Abdominal pain; Vomiting; Diarrhea Jaundice Hepatomegaly	Patient 4: 8	Patient 4:<br><br>Severe obesity<br><br>NAFLD	Patient 4: January 2021<br><br>Mild	Patient 4: 94 days after COVID-19 diagnosis	Patient 4: AST: 1551 IU/L<br><br>ALT: 2439 IU/L<br><br>ALP: 499 IU/L<br><br>GGT: 95 IU/L<br><br>TBili: 10.3 mg/dl<br>DBili: 6.2 mg/dl<br><br>Albumin: 4.2 g/dL	Patient 4: Systemic steroids<br><br>Bone marrow transplant for aplastic anemia	Patient 4: Aplastic anemia (2 months after presentation)	Patient 4: 4 months

						Ammonia: 71 µg/ dL<br><br>INR: 1.2<br><br>SARS-CoV-2 antibody positive: 376 AU/mL			
	Patient 5: Acute hepatitis with cholestasis<br><br>1st admission: Weakness; Diarrhea; Abdominal pain; Jaundice	Patient 5: 13	Patient 5: None	Patient 5: September 2021<br><br>Mild	Patient 5: 1st admission: with initial COVID-19 infection	Patient 5: 1st admission: AST: 2901 IU/L<br><br>ALT: 9376 IU/L<br><br>ALP: 396 IU/L<br><br>GGT: 141 IU/L<br><br>TBili: 12 mg/dl<br><br>DBili: 8.8 mg/dl<br><br>Albumin: 3.8 g/dL<br><br>Ammonia: 62 µg/ dL<br><br>INR: 1.2	Patient 5: 1st admission: None	Patient 5: None	Patient 5: 45 days

								SARS-CoV-2 PCR 'positive':				
			2nd admission: Vomiting; Abdominal pain; Jaundice; Hepatomegaly; RUQ Tenderness				2nd admission: 53 days after COVID-19 diagnosis	2nd admission:<br>AST: 2901 IU/L<br><br>ALT: 9376 IU/L<br><br>ALP: 396 IU/L<br><br>GGT: 141 IU/L<br><br>TBili: 12 mg/dl<br><br>DBili: 8.8 mg/dl<br><br>Albumin: 3.8 g/dL<br><br>Ammonia: 62 µg/ dL<br><br>INR: 1.2	2nd admission: systemic steroid treatment			
Ahmed et al. 2022 [15]	Case study<br><br>India	1 (100% M)	Congestive hepatopath y	17	None	June 2021<br><br>Mild COVID-19	15 days after COVID-19 diagnosis	Platelets 81,000/cumm<br>Na: 126 mg/dL	Day 1: Decongested using: Furosemide (40 mg BD) Spironolactone	1 week after discharge: Persistent	3 months Discharged	3<br><br>Murad et al.

								TBili: 3.9 mg/dL<br>indirect bilirubin: 2.8 mg/dL<br>AST: 584 U/L<br>ALT: 462 U/L<br>ALP: 95 U/L<br>pt time: 23 sec<br>INR: 1.8<br>LDH: 988 U/L	(25 mg OD)<br>Vitamin K (10 mg OD)<br><br>Day 3: Diuretic stopped<br><br>Dopamine<br><br>Day 7: Enalapril 2.5 mg BD<br>Spironolactone 25 mg OD<br>Furosemide 20 mg OD, Digoxin 0.25 mg OD<br><br>Day 10: Discharged	hyperbilirubinemia (2.3 mg/dL)<br><br>Reducing enzymes<br>AST: 30 U/L<br><br>ALT: 60 U/L<br>ALP: 98 U/L)<br><br>3 months after discharge: Asymptomatic with normal LFTs		
Sai et al. 2023 [16]	Single-center prospective observational study<br><br>India	78 (34.6%)	NR<br><br>Elevated SGOT: 40/78<br>SGPT 27/78<br><br>Ascites: 1/78	NR	NR	December 2020 – November 2021	2–6 weeks after the onset of acute COVID-19 infection	NR	IVIg: 94.9%<br><br>Steroid: 70.5%	Mortality: 1.3%	No follow-up	2<br><br>NOS
Kolesova et al. 2021 [17]	Cross-sectional, single-center study<br><br>Latvia	Acute COVID-19 group<br>66 (50% M)	NR<br><br>Liver fibrosis	NR	NR	Acute infection	At time of study	Liver fibrosis index FIB-4 > 1.45 (52%)<br><br>FIB-4 ≥ 3.25(29%)	NR	Liver fibrosis	No follow-up	9<br><br>NOS

								Hyaluronic acid HA ≥ 75 ng/ml (54%)				
		Post-COVID-19 group<br>58 (53% M)	NR<br><br>Liver fibrosis	NR	NR	NR	3–6 months after recovery	FIB-4 > 1.45 (5%)<br><br>FIB-4 ≥ 3.25 (2%)<br><br>Hyaluronic acid HA ≥ 75 ng/ml (15%)	NR	Liver fibrosis	No follow-up	
		Control group<br>17 (53% M)	NR	NR	NR	NR	NR	NR	NR	NR	No follow-up	
Daid et al. 2020 [18]	Case study<br><br>USA	1 F	100%<br><br>Intraparenchymal liver hemorrhage<br><br>Severe right upper quadrant pain and back pain	43	IDDM<br>HTN<br>HLD	ICU admission	15 days after admission	AST: 1111U/L<br><br>ALT: 1020 U/L<br><br>ALP: 203 IU/L<br><br>Normal TB	Hydroxychloroquine tocilizumab convalescent plasma		Until discharge only	5<br><br>Murad et al.
Ma et al. 2023 [19] 65	Large-scale population-based cohort study based on a prospective cohort	COVID-19 group<br>112,311 (45.2% M)	NR<br><br>Severe liver disease<br><br>Liver failure	NR	NR	COVID-19 diagnosed before 30 November 2020	30 days or more after diagnosis of SARS-CoV-2 infection or	NR	NR	NR	254 ([IQR] 184 – 366) days	8<br><br>NOS

UK		Hepatic sclerosis or cirrhosis<br><br>NAFLD			Non-hospitalized COVID-19<br><br>Hospitalized COVID-19<br><br>Severe COVID-19	index date				
	Contemporary control group 359,671 (44.6%)	NR<br><br>Severe liver disease<br><br>Liver failure<br><br>Hepatic sclerosis or cirrhosis<br><br>Complication of liver disease<br><br>NAFLD	NR	NR	NR	30 days or more after diagnosis of SARS-CoV-2 infection or index date	NR	NR	NR	254 (184 – 366) days
	Historical control group 370,979 (55% M)	Severe liver disease; Liver failure; Hepatic sclerosis or cirrhosis;	56.94	NR	NR	30 days or more after diagnosis of SARS-CoV-2 infection or	NR	NR	NR	254 (IQR 184 – 367) days

			Complication of liver disease; NAFLD				index date					
Anguiano-Albarran et al. 2023 [20]	Case report<br><br>USA	1 F	1 (100%)<br><br>Acute liver failure; Encephalopathy-diffuse jaundice; Subsequently; NAFLD	68	RA<br>Afib<br>DVT<br>HTN<br>Obesity (BMI 33.93)	10 days prior to presentation<br><br>COVID-19 pneumonia<br><br>No ICU admission	Detected on admission (10 days after initial COVID-19 symptoms)	Upon admission:<br><br>INR: 2.4<br><br>PT: 29.1 seconds<br><br>aPTT: 27.9 seconds<br><br>Ferritin: 3149 ng/mL<br><br>C-reactive protein: 12.7 mg/dL<br><br>Blood urea nitrogen: 126 mg/dL<br><br>Serum creatinine: 5.54 mg/dL<br><br>TBili: 24.9 mg/dL<br><br>DBili: 14.4 mg/dL	First 3 inpatient days: Intravenous albumin<br><br>Midodrine octreotide<br><br>Diltiazem	Superimposed COV-19<br><br>Nephropathy with concomitant prerenal azotemia secondary to dehydration	NR	3<br><br>Murad et al.

								AST: 525 IU/L<br><br>ALT: 583 IU/L<br><br>ALP: 201 U/L<br><br>Albumin: 1.3 g/dL<br><br>Lactic acid: 3.4 mmol/L<br><br>Hepatitis serology negative<br><br>Anti-mitochondrial antibody negative<br><br>Smooth muscle antibody weakly positive				
Soldera et al. 2023 [21]	Case report<br><br>Brazil	1 M	1 (100%)<br><br>Acute liver injury due to hemophagocytic lymph histiocytosis;	57	PMR	Diagnosed a month earlier<br><br>Mild COVID-19 infection	On admission; 1 month after COVID-19 diagnosis (during recovery)	HScore: 238 points (98-99% probability of HLH)<br><br>Platelets: 209,000/mm³	Etoposide<br><br>Dexamethasone<br><br>Cyclosporine (maintenance)	MRI showed enlarged: Abdominal lymph nodes Liver Spleen	NR<br><br>5 month for recovery	2<br><br>Murad et al.

			Bone marrow biopsy showed hyperplasia; Megakaryocytes dysplasia; Histiocytosis hemophagocytosis<br><br>Jaundice<br><br>High grade fever<br><br>Upper abdominal pain					AST: 473 U/L<br><br>ALT: 128 U/L<br><br>TBili: 1.5 mg/dL<br><br>Serum ferritin level above 2,000 ng/mL				
Radzina et al. 2022 [22]	Observational cohort study<br><br>Latvia	Post-COVID-19 56 (50% M)	NR<br><br>5 with increased echogenicity on US<br><br>8 with steatosis indicators on MR<br><br>8 with F1 liver fibrosis	NR	NR	1.3 ± 0.6-month duration (0.2-3 range)<br><br>Mild: 13/30 (43%)<br><br>Moderate 10/30 (33%)<br><br>Severe: 7/30 (23%)	6.4 ± 1.9 months ago (3–9-month range) after infection	NR	NR	NR	NR	5<br><br>NOS

			3 F2 fibrosis (P< .001)<br><br>2 F3-F4 fibrosis<br><br>No symptoms reported			Hospitalized for COVID-19: 34						
		Control 34 (38%)	Increased echogenicity on US: 4<br><br>Steatosis indicators on MR: 3<br><br>F1 liver fibrosis: 1<br><br>No reported symptoms	NR	NR	NR	NR	NR	NR	NR	NR	
Pesti et al. 2023 [23]	Observational cohort study<br><br>Hungary	150 (54% M)	99 (66%)<br><br>Autopsy found: Cirrhosis/<br><br>F 4 fibrosis: 7<br><br>F3 fibrosis: 3	NR	NR	Mean length of stay: 17.26 days<br><br>83 cases requiring intensive care	Length of time after COVID-19 infection NR<br><br>Postmortem time before autopsy averaged	Elevate AST > 13-40 U/L" in 57/120<br><br>Elevated ALT > 7-40 U/L" In 51/ 120<br><br>Elevated ALP > 40-130	NR		NR	6<br><br>NOS

F1/2 fibrosis: 68<br><br>No fibrosis/F0: 72<br><br>Steatosis (n=147):<br>F3: 15<br>F2: 29<br>F1: 49<br>F0: 54<br><br>Cholestasis (n=143):<br>F2: 2<br>F1: 52<br>F0: 89<br><br>Chronic inflammation n=146):<br>F2: 4<br>F1: 59<br>F0: 83<br><br>Endothelial damage (n=119):<br>F3: 46<br>F2: 52<br>F1: 21<br>F0: 0	3.5 days (0.25-17-day range)	U/L in 46/120<br><br>GGT > 12-52 U/L in 91/120<br><br>Bilirubin >5-21 μmol/L in 29/119<br><br>CRP>10 mg/L in 124/129<br><br>Low lymphocyte count <1.5-4 G/L in 98/131<br><br>Elevated neutrophil count > 2-7.5 G/L in 88/131

			Sinus dilatation (n=119):<br>F3: 31<br>F2: 52<br>F1: 36<br>F0: 0									
Liemarto et al. 2021 [24]	Case report<br><br>Indonesia	1 M	Liver abscesses with hemorrhage and parenchymal necrosis<br><br>Severe abdominal pain<br><br>Right upper abdominal tenderness<br><br>Hepatomegaly<br><br>Bloody diarrhea (4 times)<br><br>Elevated liver enzymes and hypoalbuminemia	49	DM	NR<br><br>Severe COVID-19 pneumonia admitted to ICU	1 month after infection	AST (U/L):<br>Day 7: 571<br>Day 10: 131<br>Day 12: 157<br>Day 14: 44<br>Day 20: 56<br><br>ALT (U/L):<br>D7:484<br>D10: 225<br>D12: 141<br>D14: 66 D20: 17<br><br>PT (second):<br>D7: 12.2 D10: 11.0 D16: 9.9<br><br>APTT (second):<br>D7: 47.7 D10: 31.5 D20: 33.5<br><br>Albumin (g/dl):<br>D7: 1.63 D10: 2.58 D12: 2.16 D14:	Day 1: 4L nasal oxygen<br><br>D4: Enoxaparin amikacin and imipenem-cilastatin<br><br>D7-10: Exploratory laparotomy liver abscess drainage and necrosectomy<br>4 units fresh frozen plasma Tranexamic acid Cefotaxime Metronidazole Pantoprazole Electrolytes Albumin<br><br>D10-20: Mycamin Cefoperazone Sulbactam	Died on day 20	Died on day 20	4<br><br>Murad et al.

			due to liver abscess					2.27 D16: 2.55 D20: 2.34	Moxifloxacin			
Roman et al. 2022 [25]	Prospective cohort study<br><br>Romania	78 (56% M)	58 (74%)<br><br>Abnormal LFTs and US findings<br><br>Hepatic steatosis: 20 (26%)<br><br>Gallbladder sludge: 10 (13%)<br><br>Portal venous system thrombosis : 2 (2.5%)<br><br>lymphadenopathy: 15 (19%)	NR	NR	NR<br><br>60 non-severe<br><br>18 severe	6 months after discharge	NR	NR	NR	6 months	5<br><br>NOS
Milic et al. 2022 [26]	Retrospective cohort study<br><br>Italy	235 (68.9% M)	105 (77%)<br><br>MAFLD<br><br>No symptoms reported	61 (52-72.5)		On Average COVID-19 144 days before<br><br>Average duration 11.8 days	MAFLD retrospectively estimated in 88 patients (37.3%)		NR	Respiratory musculoskeletal neurocognitive psychological sensory	NR	2<br><br>NOS

					Patients with invasive/noninvasive ventilation: 45	MAFLD measured by transient elastography in 130 patients after COVID-19 (average 145 days)			and dermatological complications<br><br>No reported Liver dysfunction outside MAFLD	
	105 (63.8% M) No MAFLD at follow-up	NR<br><br>Median reported PACS symptoms: 3	63 (52-74)	DM: 7<br>HTN: 27<br>Metabolic syndrome: 14<br>Insulin resistance: 25	On Average COVID-19 143.5 days before (p=0.19 MAFLD vs. non-MAFLD)<br><br>Average duration 11.5 (p=0.61)<br><br>Patients with invasive/noninvasive ventilation: 21 (p=.90)	as above	AST: 35.0 U/L (p=0.71 MAFLD vs. non-MAFLD)<br>ALT: 23.0 U/L (p=0.001)	NR	as above	as above
	130 (73.1% M)	NR	60 (52-70)	DM: 26<br>HTN: 43	On Average COVID-19		AST: 37.0 U/L (p=0.71 MAFLD vs.	NR	As above	As above

		MAFLD at follow-up	MAFLD criteria: Presence of liver steatosis (score>36 on hepatic steatosis index) + BMI >25 OR T2DM)<br><br>Median reported PACS symptoms: 2		Metabolic syndrome: 51<br>Insulin resistance: 60	145.0 days before<br><br>Average duration 12.1<br><br>patients with invasive/noninvasive ventilation: 24		non MAFLD)<br>ALT: 36.0 U/L<br>(p=0.001)				
Bende et al. 2021 [27]	Prospective cohort study<br><br>Romania	Pulmonary Injury Group<br>53 (43.4%)	NR<br><br>Liver stiffness<br><br>Steatosis, and viscosity<br><br>No clinical correlations were investigated<br><br>Liver stiffness- via transient elastography:	NR	No liver or cardiac comorbidities	Duration NR<br><br>13.7% TCT Pulmonary injury<br><br>4 symptoms of COVID-19	Initial evaluation 7 weeks since COVID-19 infection	NR	NR	NR	NR	5<br><br>NOS

		5.08 ± 1.40 (kPa)<br><br>Viscosity via US: 1.74 ± 0.28 (PaS)<br><br>Sound speed via US: 1530.3 ± 24.91 (m/s)								
	Non-pulmonary injury group 44 (31.8%)	NR<br><br>Liver stiffness- via transient elastography: 4.39 ± 1.41 (kPa)<br><br>Viscosity via US: 1.64 ± 0.25 (PaS)<br><br>Sound speed via US:1542.88 ± 27.66 (m/s)	NR	NR	Duration NR<br><br>0% TCT Pulmonary injury<br><br>2 symptoms of COVID-19	Initial evaluation 8 weeks since COVID-19 infection	NR	NR	NR	NR

Shorbagi et al. 2023 [28]	Case report<br><br>UAE	1 (100%)	1(100%)<br><br>Autoimmune hepatitis<br><br>Epigastric pain radiating to the back, nausea, and vomiting + 2 months of diarrhea and 2-3 weeks of bloody stools	33	None	January 2022<br><br>Self-reported to be mild. Duration NR	2 months after recovery from infection	AST: 76 U/L<br><br>ALT: 119 U/L<br><br>Antinuclear antibodies (ANA): 1:1000 titer<br><br>Anti-smooth muscle antibodies: 65U<br><br>GGT: 45 U/l<br>ALP : 59 U/l	60 mg prednisolone IV<br><br>Long term: mesalamine azathioprine	Pancreatitis and UC diagnosed during this hospitalization<br><br>UC correlates to Primary Sclerosing Cholangitis - this was ruled out by MRCP	7 months after hospitalization Patient is asymptomatic	5<br><br>Murad et al.
Lai et al. 2023 [29]	Case report<br><br>Taiwan	1 F	1 (100%)<br><br>Acute liver injury<br><br>Calcified liver nodule<br><br>No clinical symptoms of hepatitis	60	None	12 July, 2022<br><br>Mild	Day 39 after COVID-19 diagnosis.<br><br>After recovery from infection	Day 39<br><br>AST:207 U/L<br><br>ALT:570 U/L<br><br>TBili: 1.0mg/dL<br><br>ALP:87 U/L<br>GGT:84 U/L<br><br>No evidence for acute Hep A,B or C.	expectant management	None	Over 3 months<br><br>LFT trended down until returning to normal by the last follow-up visit	4<br><br>Murad et al.
Daga et al. 2021 [30]	Case report<br><br>India	1 F	1 (100%)	25	NR	19 September 2020	About 1 month	Labs at peak	Prednisolone at 40 mg/d for 2 weeks	3 months after reso-	5 months after	6<br><br>Murad et

		Acute hepatitis<br><br>Dark urine nausea abdominal pain<br><br>Acalculous cholecystitis<br><br>Autoimmune hemolytic anemia<br><br>Jaundice nausea abdominal pain<br><br>Mild hepatomegaly (15.0 cm)			Mild	after pulmonary symptoms subsided.	DBili: 34.32 mg/dL<br><br>AST: 682 mg/dL<br><br>ALT: 516 mg/dL<br><br>ALP: 160 mg/dL		lution, patient tested positive for CMV and EBV IgG	presentation.	al.

Afib: atrial fibrillation, **ALT**: alanine aminotransferase, **ANA**: Anti-nuclear Antibodies, **APOLT**: Auxiliary partial orthotopic liver transplantation, **AST**: aspartate aminotransferase, **CAD**: Coronary Artery Disease, **CBC**: complete blood count, **CHF**: Chronic Heart Failure, **CKD**: Chronic kidney disease, **CLD**: Chronic Liver Disease, **CRP**: C-reactive protein, **COPD**: Chronic Obstructive Pulmonary Dysfunction, **DBili**: Direct Bilirubin, **DM**: Diabetes Mellitus, **DVT**: deep venous thrombosis, **ESRD**: End Stage Renal Disease**, FIB-4**: Liver Fibrosis Index, **GGT**: Gamma-glutamyl transpeptidase, **HDL**: high-density lipoprotein, **HF**: Heart Failure, **HLD**: hyperlipidemia, **HTN**: Hypertension, **LDL**: low-density lipoprotein, **IDDM**: insulin Dependent Diabetes Mellitus, **INR**: international normalized ratio, **LDH**: lactate dehydrogenase, **LFTs**: Liver Function Tests, **MAFLD**: Metabolic-Associated Fatty Liver Disease, **MI**: Myocardial Infarction, **MS**: Multiple Sclerosis, **NAFLD**: Non-alcoholic Fatty Liver Disease (now **MASLD**: Metabolic Associated Liver Disease), **PACS**: Post-Acute COVID-19 Syndrome, **PLD**: Parenchymal Liver Disease, **PVD**: Peripheral Vascular Disease, **RA**: Rheumatoid arthritis, **SGOT**: serum glutamic-oxaloacetic transaminase, **SGPT:** serum glutamic-

pyruvic transaminase, **T1DM**: Type 1 Diabetes Mellitus, **T2DM**: Type 2 Diabetes Mellitus, **TBili**: Total bilirubin, **US**: Ultra Sound, **QoL :** quality of life, **PMR**: Polymyalgia rheumatica.
